# Socorristas en red: Soporte comunitario a la autogestión del aborto en Argentina

**DOI:** 10.18294/sc.2024.4810

**Published:** 2024-06-20

**Authors:** Gabriela Luchetti, Valeria Albardonedo, María Victoria Alfonso

**Affiliations:** 1 Médica, ginecóloga-obstetra. Magíster en Genero Sociedad y Políticas. Profesora adjunta, Facultad de Medicina, Universidad Nacional del Comahue, Neuquén, Argentina. gluchetti56@gmail.com Universidad Nacional del Comahue Facultad de Medicina Universidad Nacional del Comahue Neuquén Argentina gluchetti56@gmail.com; 2 Licenciada en Comunicación Social. Magíster en Ciencias Sociales. Asistente Docencia, Facultad de Medicina, Universidad Nacional del Comahue, Neuquén, Argentina. valealbar@gmail.com Universidad Nacional del Comahue Facultad de Medicina Universidad Nacional del Comahue Neuquén Argentina valealbar@gmail.com; 3 Licenciada en Comunicación Social. Magíster en Política y Gestión de la Ciencia y la Tecnología. Secretaria, Consejo Superior, Universidad Nacional del Comahue, Neuquén, Argentina. alfonsomariavictoria@gmail.com Universidad Nacional del Comahue Universidad Nacional del Comahue Neuquén Argentina alfonsomariavictoria@gmail.com

**Keywords:** Aborto Legal, Aborto Inducido, Misoprostol, Redes Comunitarias, Feminismo, Argentina, Legal Abortion, Induced Abortion, Misoprostol, Community Networks, Feminism, Argentina

## Abstract

La disponibilidad de medicamentos para producir un aborto, sobre todo en contextos de acceso restringido, transformó las prácticas y permitió que las propias mujeres y/o sus organizaciones comunitarias ayuden a otras mujeres a abortar, interactuando o no con el sistema de salud. Este estudio recupera la experiencia de una organización feminista de la comunidad que, desde la provincia de Neuquén, se extiende a todo el país, generando una red de cuidados comunitarios. Se realizó un estudio exploratorio descriptivo, con enfoque cualitativo con el propósito de analizar las experiencias de las mujeres que facilitan el acceso al aborto permitido en Argentina. A través de entrevistas en profundidad a tres líderes de la colectiva feminista La Revuelta y de entrevistas semiestructuradas a 33 integrantes de las grupas socorristas, realizadas entre noviembre de 2019 y diciembre de 2020, describimos su historia y los procesos de trabajo y crecimiento; exploramos sus motivaciones y sentimientos y caracterizamos las interacciones de dichas organizaciones con los sistemas de salud público y privado. Los resultados de este trabajo coinciden con la conversación y la producción bibliográfica internacional acerca de estas organizaciones y sus particularidades y con la necesidad de incorporar estos cuidados a los sistemas de salud institucionales.

## Abstract (en)

The availability of medications to induce abortion, especially in contexts of restricted access, has transformed practices and allowed women and/or their community organizations to assist other women in obtaining abortions, whether or not they interact with the healthcare system. This study recovers the experience of a feminist community organization that, from the province of Neuquén, extends throughout the country, creating a network of community care. An exploratory descriptive study with a qualitative approach was conducted to analyze the experiences of women who facilitate access to permitted abortion in Argentina. Through in-depth interviews with three leaders of the feminist collective La Revuelta and semi-structured interviews with 33 members of the socorrista groups, conducted between November 2019 and December 2020, we describe their history and processes of work and growth; we explore their motivations and feelings and characterize the interactions of these organizations with public and private health systems. The results of this work align with the international conversation and bibliographic production about these organizations and their particularities, and with the need to incorporate these forms of care into institutional health systems.

## INTRODUCCIÓN

El aborto inseguro es un problema altamente prevalente e inequitativo y un grave problema de salud pública en muchos países, sobre todo en aquellos en los que existen restricciones legales simbólicas e institucionales. El aborto inducido fue definido por la Organización Mundial de la Salud (OMS) en 1993 como “inseguro” cuando es realizado por personas sin las habilidades necesarias y/o en un medioambiente que no reúne los estándares médicos necesarios^1^. Actualmente, la difusión y uso del aborto inducido con medicamentos pone en cuestión la definición de aborto inseguro^2^. La generalización del uso de medicamentos para abortar ha mejorado el acceso a abortos autoinducidos o con apoyo de proveedores no médicos, tendencia que ha producido un cambio en su categorización. Ya no resulta adecuado calificar un aborto como inseguro, únicamente por el tipo de proveedor o por el medio ambiente en que este se realiza^3^.

Asegurar el bienestar y la efectividad de un procedimiento de aborto inducido requiere algo más que un operador calificado y un procedimiento apropiado, demanda que sea seguro, que las mujeres no corran el riesgo de recibir sanciones por tomar la decisión de interrumpir su embarazo o ser estigmatizadas por ello^4^.

Para una atención de calidad debe existir una gradación que contemple quiénes, con qué y dónde se produce la atención, además del clima legal y social en donde esto ocurre^5^. Desde 1921, el Código Penal Argentino establece excepciones para considerar legal un aborto, estas son: la violación y el riesgo para la salud o la vida de la persona gestante. Sin embargo, nuestra historia muestra que, aún para las excepciones legales, el aborto ocurría en el circuito clandestino y en forma insegura. Antes de su legalización en diciembre del 2020, dos hechos habilitaron la práctica menos restrictiva del aborto en nuestro país: el fallo “F.A.L.” de la Corte Suprema de Justicia (CSJ) y la existencia del aborto con medicamentos.

El fallo “F.A.L.” de la CSJ de 2012 dio lugar a lecturas no restrictivas del código penal y estableció los alcances de las excepciones para realizar un aborto. La existencia de medicamentos para abortar transformó las prácticas, permitiendo a las mujeres abortar en forma segura por fuera del sistema de salud formal^6^. 

Los medicamentos para abortar y el conocimiento sobre ellos permite que el aborto sea más temprano, más accesible, más seguro, menos traumático, menos medicalizado y más económico^7^^,^^8^^,^^9^. La aceptabilidad por las mujeres y los proveedores es alta, así como su seguridad y efectividad^10^^,^^11^.

Con estos antecedentes, durante el año 2012, en la ciudad de Neuquén, la colectiva feminista La Revuelta inicia una línea telefónica de ayuda para mujeres que deseaban abortar, llamada “socorro rosa”, con el propósito de proveer información y acompañamiento durante el procedimiento del aborto con medicamentos. En el año 2014, como parte de un plan de acción de La Revuelta, esta experiencia se extiende a todo el país, con la fundación de “Socorristas en Red” (SenRed).

El propósito de este estudio es analizar las experiencias de las organizaciones de mujeres que, desde prácticas feministas colectivas en red facilitan el acceso al aborto en Argentina, con el objetivo de describir su historia y los procesos de trabajo y crecimiento; explorar sus motivaciones y sentimientos; y caracterizar las interacciones de dichas organizaciones con los sistemas de salud público y privado, que produjeron transformaciones observables en el acceso al aborto en nuestro país^12^^,^^13^, poniendo en valor el poder y el virtuosismo de las acciones comunitarias en salud.

## METODOLOGÍA

Se llevó a cabo un estudio exploratorio-descriptivo con enfoque cualitativo. Se definió una muestra no probabilística por conveniencia que tuvo por objetivo representar los distintos grupos de Socorristas en Red de acuerdo a su ubicación geográfica. Las personas entrevistadas fueron contactadas con un primer dato proporcionado por una líder de La Revuelta y a partir de allí mediante la técnica bola de nieve. El contacto inicial se realizó a través de la aplicación WhatsApp. Se enviaba el documento informativo para dar el consentimiento inicial y se ofrecía más información al comienzo de la entrevista en línea, y se solicitaba el consentimiento verbal para entrevistar y grabar. 

Entre los meses de noviembre de 2019 y diciembre de 2020, se realizaron entrevistas en profundidad a tres informantes claves de organización La Revuelta de Neuquén, creadora y pionera en el accionar del dispositivo; y entrevistas semiestructuradas a 33 activistas de las organizaciones feministas que integran la agrupación Socorristas en Red. Cada una de las activistas representaba un nodo diferente de la red, llamado *grupa* (feminización de la palabra “grupo”, usada por las socorristas corrientemente para referirse a cada agrupación, uso adoptado en este texto). 

La muestra quedó conformada con un 68,5% de las grupas existentes en todo el país y corresponden a las provincias de Chubut, Neuquén, Río Negro, Tierra del Fuego, La Pampa, diversas regiones de la provincia de Buenos Aires, Entre Ríos, Córdoba, Misiones, Corrientes, Chaco, Formosa, Salta, Jujuy, Catamarca, San Juan, San Luis, La Rioja, Ciudad Autónoma de Buenos Aires y Santa Fe. Hay agrupaciones en 21 provincias argentinas y solo en Mendoza y Santa Cruz no existen. En Tucumán y Santiago del Estero no se hicieron entrevistas por falta de respuestas a la convocatoria. 

Las edades de las entrevistadas oscilaron entre 24 y 56 años con una edad promedio de 35 años. Salvo una de las 33 activistas, todas tenían otra ocupación y/o profesión además de dedicar tiempo a realizar su tarea de acompañar a las mujeres en sus procesos de aborto (Tabla 1). 

**Tabla 1:** Perfil de las socorristas. Argentina, noviembre de 2019 a diciembre de 2020.

Provincia	Edad	Ocupación/profesión	Nombre de la Grupal
Buenos Aires	35	Docente	Socorro Rosa Necochea Quequen
Buenos Aires	36	Empleada	Bahia Rosa
Buenos Aires	49	Doula	Manos a la otra
Buenos Aires	41	Docente	Socorro Rosa
Buenos Aires	35	Enfermera	Brasas
Buenos Aires	38	Empleada administrativa	Las Elviras
Buenos Aires	49	Empleada administrativa	Las Bravas
CABA	40	Empleada administrativa	La Revuelta CABA
Catamarca	39	Fotógrafa	Socorristas Catamarca
Chaco	31	Diseñadora	Resistencia Rosa
Chubut	30	Estudiante	Socorro Rosa Rabiosa
Córdoba	52	Docente	Córdoba Hilando
Corrientes	32	Docente	Enredadera Rosa
Entre Ríos	26	Desocupada	Socorristas Costa del Uruguay
Formosa	36	Docente	Lapacha Rosa
Jujuy	26	Artista	Socorro Rosa Jujuy
La Pampa	37	Docente	Socorro Santa Rosa La Pampa
La Rioja	27	Estudiante	Socorro Rosa La Rioja
Misiones	29	Comerciante	Socorristas Corrientes
Neuquén	25	Estudiante	La Revuelta
Neuquén	51	Docente	La Revuelta
Neuquén	33	Docente	La Revuelta
Neuquén	56	Docente	La Revuelta
Neuquén	56	Docente	La Revuelta
Río Negro	42	Docente	Maleducadas-Kisuelain
Río Negro	40	Trabajadora social	La Revuelta
Río Negro	33	Socióloga	Socorro Fiske
Río Negro	33	Enfermera	Rosa Salvaje Viedma
Río Negro	40	Trabajadora social	Socorro Villa Regina
Río Negro	56	Docente	Comarca Andina
Salta	29	Docente	Las Bomberas
San Juan	33	Docente	Las Hilarias
San Luis	55	Comerciante	Las Rudas
Santa Fe	24	Estudiante	Las Nanas
Tierra del Fuego	30	Empleada administrativa	Peste Rosa

Se asignó un número único de identificación a cada entrevistada. Este número (iniciales de nombre y apellido y día de la semana y mes de la entrevista) sirvió como identificador de la participante en el estudio, en la base de datos del estudio y para almacenar los datos recopilados de cada persona participante. Solo el equipo de investigación podía vincular los datos del estudio con el participante a través de una lista de identificación archivada en la Facultad de Ciencias Médicas, de la Universidad Nacional del Comahue. Se respetaron las regulaciones de privacidad y protección de datos al recolectar, enviar, procesar y almacenar datos personales individuales. Todos los datos recopilados en línea se almacenaron en la plataforma virtual. Se dispuso de un sitio con un archivo para la coordinación al inicio del estudio. Este archivo contiene los documentos y está disponible para revisión y monitoreo durante y después del estudio. 

En el contexto de este estudio se decretó el Aislamiento Social Preventivo y Obligatorio (ASPO) por la pandemia de covid-19^14^^)^ y se aprobó la Ley de Interrupción Voluntaria del Embarazo (Ley IVE 27610), en diciembre de 2020. Ambas situaciones fueron consideradas en las entrevistas. 

Las entrevistas en profundidad realizadas a las integrantes de la colectiva feminista La Revuelta de Neuquén, quienes crearon el dispositivo “Socorro Rosa” y más tarde la red nacional Socorristas en Red, estuvieron orientadas a relevar la historia de la agrupación y los procesos de nacionalización del dispositivo “Socorro Rosa”. Las entrevistas semiestructuradas a las activistas integrantes de las distintas agrupaciones de Socorristas en Red se ordenaron en temas y subtemas, y estuvieron orientadas a conocer las motivaciones para unirse a una agrupación, a la descripción de los procesos seguidos en el dispositivo “Socorro Rosa”, las percepciones y sentimiento de las activistas en la tarea y a las relaciones con el sistema de salud formal.

El equipo de investigación estuvo conformado por tres personas que fueron quienes realizaron el total de las entrevistas. Una vez transcriptas, se realizó un análisis temático de los datos apoyados por el software Atlas-Ti 9. Se siguieron las etapas de codificación, definición de temas y subtemas y se elaboró un árbol jerárquico. Se realizó una relectura e interpretación de los resultados iniciales. 

El proyecto fue evaluado y aprobado por la Comisión Asesora en Investigación Biomédica en Seres Humanos de la provincia de Neuquén (CAIBISH) (Resolución 02025 junio 2019, Subsecretaría de Salud, Provincia de Neuquén).

## RESULTADOS

Para dar mayor claridad presentamos los resultados principales del estudio en dos partes. En primer lugar, se presentan las entrevistas en profundidad a las líderes de La Revuelta, como relatoras del camino que inicia, difunde y sostiene la red de acompañantes de abortos autogestionados, organizados en tres ejes: 1) La Revuelta: Historia e hitos fundantes; 2) Acompañar colectivamente: el dispositivo “Socorro Rosa” y sus relaciones con el sistema de salud; y 3) Difundir y crecer: La red nacional “Socorristas en Red”.

En segundo lugar, se presentan las entrevistas a las integrantes de las grupas socorristas que dan cuenta de los procesos del socorrismo, las relaciones con el sistema de salud formal y sus propias sensaciones y motivaciones en la tarea, que se abordan en los siguientes ejes: 1) Cuidar colectivamente: las motivaciones de las Socorristas en Red; 2) La tarea de acompañar colectivamente; 3) Percepciones, sentimientos y satisfacción con la tarea; 4) Relación con el sistema de salud (público y privado) y sus proveedores; 5) La pandemia; y 6) El socorrismo y la Ley de IVE/ILE.

### Líderes de la colectiva feminista La Revuelta

#### La Revuelta: Historia e hitos fundantes

La idea de fundar una agrupación feminista nace en el encuentro de mujeres del año 2000. La idea y el entusiasmo proviene del encuentro y se plasma el 8 de marzo del año siguiente, con la creación de la colectiva feminista La Revuelta, en Neuquén capital:

“*El interés por la política y el interés por la intervención, no solo por la política para mirarla y opinarla, sino por intervenir*”. (R. Neuquén)

Las entrevistadas coinciden en señalar como hito, en la consolidación de la colectiva, su ingreso en la Campaña Nacional por el Derecho al Aborto Legal Seguro y Gratuito en el año 2005:

“*El ingreso a la Campaña implica un salto político en La Revuelta, porque nos pone ante la posibilidad de relacionarnos de manera mucho más directa con un montón de colectivas, y también empezamos a ser miradas*”. (R. Neuquén)

Se señalan otros hitos como la inauguración de un dispositivo de consejería legal para casos de violencia de género:

“*La creación del ‘Socorro violeta’ porque también nos puso a discutir algunas cosas con el Estado, en términos de interpelaciones desde esta nuestra experiencia de acompañamiento en temas de violencias*”. (M. Neuquén)

#### Acompañar colectivamente: el dispositivo “Socorro Rosa” y sus relaciones con el sistema de salud

El “Socorro Rosa” es un dispositivo generado a fines del año 2010 en la provincia de Neuquén para dar respuestas a mujeres que deseaban interrumpir su embarazo. Nació como un servicio telefónico de información sobre el aborto con medicamentos y fue más allá de la información de cómo obtener y usar la medicación. El dispositivo devino “acompañamiento” durante el proceso de aborto para contener, dar seguridad y confianza a la situación de temor, dolor e incertidumbre, en un contexto en el que, además de percibirse ilegal, el aborto estaba construido socialmente y desde el discurso médico como una práctica peligrosa. 

El ingreso de La Revuelta a la Campaña Nacional por el Derecho al Aborto Legal Seguro y Gratuito en el año 2005 generó un espacio de consultas telefónicas espontáneas acerca de a quién recurrir en situaciones de aborto. Estas consultas se hacían a la línea pública que había sido creada para dar consejería legal sobre violencia de género. A través de esta línea, las activistas comenzaron a informar sobre la existencia de médicos que, por ese entonces, realizaban abortos quirúrgicos en la región:

“*Por ser parte de la campaña, muchas personas nos empezaban a llamar y a preguntar cómo resolver situaciones de aborto*”. (B. Neuquén)

“*En ese momento, derivamos a personas que nos contactaron, les decíamos ‘mirá, sabemos que hay un médico que trabaja en tal lugar’. Pero no conocíamos al médico*”. (M. Neuquén)

Sin embargo, rápidamente las activistas notaron que esas mujeres eran maltratadas de múltiples formas durante esas experiencias clandestinas, poco seguras y pagas:

“*Ya no se podía más hacer eso de pasar el dato de los médicos porque claramente no era una práctica cuidada y segura en ese momento, y ahí empezamos a leer sobre misoprostol, sobre oxaprost, y empezamos a pasar ese dato, como ‘podés comprarlas acá y se usan de esta manera*’”. (M. Neuquén)

De esa manera, basándose en la experiencia local de lesbianas y feministas por la descriminalización del aborto, en su publicación “Todo lo que querés saber sobre cómo hacerse un aborto con pastillas”^15^ comenzaron a dar otras respuestas y generaron el dispositivo “Socorro Rosa”:

“*Usamos el libro de las lesbianas y feministas, usamos algunas partes de ese libro para un folleto propio que habíamos hecho que después reformulamos y a partir de todo el cúmulo de conocimiento que fuimos teniendo por los propios acompañamientos, hicimos nuestro propio folleto y dejamos de usar esos materiales, pero al principio sí fue una base muy importante para nuestro conocimiento, para acompañar digamos*”. (B. Neuquén)

En ese armado del dispositivo consideraron fuertemente la articulación con el sistema de salud “formal”:

“*Entonces, siempre el interés fue trabajar con el sistema de salud, sobre todo el público, pero también el privado, y nos dedicamos mucho a eso*”. (B. Neuquén)

Las primeras estrategias devienen de la necesidad de contar con recetas de misoprostol:

“*Después empezamos a notar que se les dificultaba la compra de la medicación, porque le pedían receta, por ejemplo. Entonces empezamos a pedirle a nuestras amigas médicas*”. (R. Neuquén)

Desde el inicio de las actividades del dispositivo, las activistas llevan un registro de datos de cada uno de estos procedimientos de acompañamiento, y en el procesamiento de estos datos surge que un gran número de mujeres que recurrían a la línea telefónica eran enviadas por médicos:

“*Los médicos les decían: ‘yo no te puedo hacer ni la receta ni te puedo hacer un aborto, pero andá a ver a estas chicas que capaz te pueden ayudar*’”. (M. Neuquén)

Esto despierta la necesidad de conocer y articular con estos actores. Para ello, idearon una ingeniosa estrategia: solicitaban turno con esos profesionales, como para recibir atención y ya en la consulta, les revelaban quiénes eran y tenían una conversación en cuanto a las articulaciones posibles. En general, eran bien recibidas por esos profesionales:

“*Sacábamos turnos personales y nos sentábamos y les decíamos: ‘soy B. de La Revuelta y vos nos mandás mujeres y queremos que nos conozcas’. Y le decíamos: ‘¿Podemos mandarte a algunas mujeres para que vos hagas el control post aborto?’. ‘Sí, por supuesto, las que yo les mando que vuelvan, y si necesitan de alguna otra, no hay problema*’”. (B. Neuquén)

Otra de las experiencias iniciales de articulación de La Revuelta con el sistema público de salud fue el consultorio Te Acompañamos (TeA) creado en el servicio de ginecología del Hospital Castro Rendon (hospital de máxima complejidad provincial, ubicado en la ciudad de Neuquén). Se trataba de un consultorio de demanda espontánea para realizar cuidados, consejería y anticoncepción en el posaborto de las mujeres que lo requirieran:

“*Después la G. dijo: ‘Bueno listo, hay que armar un espacio’, bueno, se armó el espacio, pero porque ya era una demanda super alta. Pasábamos de una o dos a tener un montón, entonces fuimos así como empujando el sistema para que se acomodara como a lo que estaba viniendo*”. (B. Neuquén)

El consultorio TeA se extendió por los centros de salud y otros hospitales:

“*Porque fui corta en lo que dije de las articulaciones con salud, también en nuestra articulación hay como distintos modos de alguna manera de acompañar por parte del sistema de salud, hay quienes garantizan el derecho, hay quienes llegan a dar la información, hay quienes dicen que no pueden acompañar, pero dan nuestro dato*”. (M. Neuquén)

Se relatan también instancias de intercambio y capacitación también con los residentes de medicina general de Neuquén en talleres y actividades conjuntas e intercambio de saberes y experiencias:

“*Lo que hacemos todo el tiempo es intentar enseñar eso que estamos aprendiendo y es como una lógica que tenemos incorporada, entonces todo el tiempo queremos mostrar eso que aprendimos a hacer y queremos enseñarlo para que otras personas también aprendan a hacerlo*”. (R. Neuquén)

#### Difundir y crecer: La red nacional Socorristas en Red

Simultáneamente con estos procesos de inicio y organización de los acompañamientos socorristas, La Revuelta comenzó a difundir lo aprendido en otras regiones del país. Entre otras acciones, llevaron al Encuentro Nacional de Mujeres del año 2013, los afiches de usos seguros del Misoprostol y desarrollaron también un taller, sobre el mismo tema. Esto produjo que muchas de las asistentes de varias provincias se acercaran, manifestando su deseo de activar su militancia del mismo modo:

“*…una de las decisiones políticas tenía que ver con intervenir y empezar a visibilizarse como socorrista*s”. (R. Neuquén)

“*Hicimos talleres. Y en esos talleres aparecieron compañeras de otros lugares del país, por ejemplo, Entre Ríos, Tucumán, de muchas provincias a querer sumarse también al Socorro Rosa*”. (M. Neuquén)

Así va surgiendo la idea de armar una red nacional de socorristas, que se inicia en 2012 y se plasma en el año 2014.

### Las colectivas integrantes de Socorristas en Red

#### Cuidar colectivamente: Las motivaciones de las Socorristas en Red

Las colectivas integrantes de Socorristas en Red (SenRed) son diversas, tanto en su conformación y sus motivaciones como en sus características socioculturales y políticas. Sin embargo, en relación con la actividad específica para llevar adelante el dispositivo “Socorro Rosa” hay acuerdos políticos específicos que hacen a la semejanza del proceso, independientemente del lugar en donde este ocurra. 

Ante la exploración de las motivaciones de las activistas socorristas para sumarse a una agrupación y realizar una actividad con información específica y acompañamiento en situaciones de aborto, surgen dos motivos principales, muy frecuentes y relacionados: haber tenido una experiencia de aborto y tener una militancia feminista y política.

La vinculación entre las diversas militancias y activismos y los abortos en sus cuerpos, no son cosas separadas, sino que en la mayoría de ellas se imbrican ambas motivaciones. En algunos casos cuentan la experiencia de un aborto vivida como muy desagradable y se unen al socorrismo como una manera de evitar que estas situaciones sean padecidas por otras mujeres. Las reflexiones que les propone su propio aborto están relacionadas con no tener información y con los temores que enfrentaron. Surge la necesidad de saber más y difundir esos saberes. El contexto de estigma, el secreto, la soledad, el desamparo y la falta de información en que estos eventos ocurrieron, fueron resaltados por las entrevistadas como vemos a continuación:

“*En mi escuela ya se había muerto la hija de la portera por un aborto, yo no sabía nada de eso, nada. Y junté ese sufrimiento, tres muertes por abortos con sonda, de esos abortos vergonzantes que vas al sepelio, y nadie dice nada*”. (M. Neuquén)

“*Lo pase muy mal, me decían ‘te podés morir’ y yo prefería morir antes que gestar*”. (D. La Rioja)

“*Fueron en el sistema privado, tuve que pagar mucho dinero. Bueno, voy a trabajar en esto para que ninguna mujer viva la soledad, sobre todo, ¿no? El aislamiento, la soledad, el silencio*”. (M. San Luis)

La situación de haber tenido un aborto acompañadas por socorristas también se suma a los motivos que esgrimieron algunas entrevistadas como fundamento para unirse a SenRed:

“*Esto se tiene que multiplicar, tengo que poder aportar algo*”. (A. Neuquén)

“*En ningún momento yo sentí que tenía que decirle las razones por que estaba tomando la decisión que estaba tomando y eso fue lo mejor*” (M. Buenos Aires)

“*Quería hacer algo más que marchar y salir a la calle*”. (M. Catamarca)

En la decisión de iniciarse en el socorrismo también influyeron los Encuentros Nacionales de Mujeres y la Campaña Nacional por el Derecho al Aborto Legal, Seguro y Gratuito, además de la presentación y debate en el congreso del proyecto de ley en 2018, así como también la pertenencia a diversos activismos feministas, gremiales, en movimientos sociales y político partidarios.

#### La tarea de acompañar colectivamente

El dispositivo “Socorro Rosa” es un servicio de asistencia a personas gestantes que desean abortar, tiene cuatro momentos claves: la llamada telefónica, el taller, el acompañamiento y el posaborto. Este proceso es seguido por las distintas grupas socorristas, es muy parecido en todas las regiones del país y cuenta con acuerdos básicos de procedimientos entre ellas. Sin embargo, como señala una entrevistada, muchas grupas presentan particularidades locales relacionadas con cuestiones históricas, culturales, religiosas, organizacionales, políticas, técnicas, de infraestructura, geográficas, etc.:

“*Las presencias de las colectivas socorristas en cada provincia son distintas y tienen que ver, por un lado, con cómo se han conformado esas colectivas, con qué individualidades. Cómo convivir en una Red que sabemos que… donde tenés que aprender que no todas van a actuar de igual manera que vos… Yo aprendo tanto, hasta aprendo de los ritmos provinciales, si no hubiéramos aprendido a convivir con esas cosas no habría Red*”. (R. Neuquén)

Cada agrupación debe tener una línea telefónica. El acuerdo político nacional es que el número debe ser público y ampliamente difundido. Es donde se establece el primer contacto y se considera un momento clave por el alto compromiso emocional que implica:

“*Esos números están publicados en toda la red en toda Argentina, para que las personas puedan llamar y acceder a información segura*”. (M. Río Negro)

Otro de los acuerdos explícitos es que se interactúa con la persona que va a abortar, tal como expresan las entrevistadas:

“*Se tiene que contactar la mujer, quien atiende tiene que hablar con la mujer que va a abortar. Si bien por primera vez a veces llama una amiga, llama la madre, la hermana, la pareja, pero en algún momento tenemos que hablar con esa mujer*”. (A. Neuquén)

“*Necesitamos saber que es una decisión que está tomando ella, la telefonista muchas veces detecta situaciones violentas*”. (L. Buenos Aires)

La llamada, cumple varios objetivos, como programar el encuentro llamado “taller”, dar información y calmar la ansiedad inicial de quien llama:

“*Pero lo que lleva al encuentro cara a cara, que se haga realidad el taller, es a través de la línea telefónica*”. (B. Río Negro)

“*En ese primer contacto se calman los miedos se genera un vínculo de confianza. Se calman ansiedades*”. (M. Río Negro)

El taller es un encuentro grupal presencial, donde se busca promover lo colectivo, quitarle la carga de culpa y estigma a la decisión de abortar, y valorar la compañía y el cuidado. A su vez, es un momento de intercambio intenso de información. 

Antes del aislamiento por la pandemia, los talleres se hacían en sitios públicos, plazas, confiterías, clubes, centros culturales, gremios y otras organizaciones de la comunidad que prestan sus espacios para el encuentro, y los días, horas y frecuencia de los encuentros se organizaban sobre la base de las posibilidades de cada agrupación.

En esa instancia de taller se aporta información concreta con todos los pasos del aborto según dos folletos impresos, basados en la guía clínica de la Organización Mundial de la Salud 2012 (cabe aclarar que era la guía vigente al momento de las entrevistas). Allí se abordan todos los detalles del uso del misoprostol y se describen los síntomas esperables durante el proceso, los síntomas colaterales y su tratamiento, así como los signos de alarma para recurrir a un centro asistencial. Se profundiza la información sobre situaciones personales y organizativas acerca de cómo y dónde tienen pensado realizar el procedimiento de interrupción y se hacen arreglos con quienes se encargarán del “acompañamiento”.

Lo grupal genera que se compartan experiencias y un sentimiento de no estar solas en esa situación. El aporte de los talleres aparece muy rico, en términos de prácticas feministas, colectivas, liberadoras y revolucionarias. Las siguientes afirmaciones fueron generalizadas, es decir, son representativas del decir de la mayoría de las entrevistadas en las descripciones del “taller”:

“*Lo más importante del dispositivo es que nos encontramos mujeres de distintas trayectorias, edades, de diferentes pertenencias sociales, vinculaciones con la maternidad y motivos por los cuales abortar y nos encontramos ahí en un plano de posibilidad de hablar del aborto*”. (G. Río Negro)

“*No nos encontramos ahí a hablar de cualquier práctica social, sino una práctica que tiene como el estigma, digamos, de estar penalizado*”. (G. Río Negro)

“*Van a abortar sin sentirse juzgadas, y no jerarquizar y que no haya un motivo que esté más ponderado que otro, como ‘un buen motivo para abortar’, y yo creo que ese es un trabajo socorrista muy interesante*”. (D. La Pampa)

“*Prepandemia nos anotábamos en tres o cuatro talleres semanales para darlos en plazas y centros culturales, entonces cada persona que llamaba podía elegir en que día como de qué horario le quedaba más cómodo para venir al taller*”. (M. Río Negro)

“*Y en ese taller leemos el folleto, tratamos de que esa lectura sea una lectura compartida, que no sea una lectura tipo transmisiva de la dupla socorrista, porque vamos de a dos, lee el folleto y hay alguna duda, no, chau. Sino que tratar de darle la palabra, si alguna quiere leer, ir haciendo partes, preguntando, hay cosas, hay detalles que es necesario remarcarlos porque el folleto es bien sencillo, no tiene ninguna complicación*”. (N. Chubut)

“*Básicamente lo que queremos es que reciba esa información, porque sabemos que cuando reciben la información se siente más seguras, y bueno, obviamente que se sienta acompañada sabiendo de que no está sola y que somos muchísimas acompañándola*”. (N. Formosa)

En la etapa de aislamiento preventivo y obligatorio, las socorristas se vieron obligadas a reconfigurarse y a reemplazar el espacio del taller como actividad grupal, los talleres se hicieron virtuales y en general con una sola persona, siempre dependiendo de las posibilidades de conectividad. El llamado telefónico pasó a tener mayor relevancia y se generó una interacción más estrecha con profesionales del sistema de salud. La actividad de las socorristas quedó más vinculada al acompañamiento y al diseño de las estrategias para el momento del aborto:

“*Ahora en pandemia, por ahí la información es más de la medicación. Cruzamos el acompañamiento con la médica y van y ella les explica, es bueno ‘¿entendiste cómo te explicó la médica, tenés alguna duda de eso?’ y bueno, ya después fluye lo otro, digamos*”. (E. Buenos Aires)

Luego de la llamada y el taller, las personas usan la medicación según las indicaciones recibidas y se lleva a cabo el proceso en el lugar con la compañía, el horario y las circunstancias que ellas han elegido. Cada persona que va a seguir el proceso de aborto se va del taller con el teléfono de la socorrista que la va a acompañar. Algunas de las descripciones señalan:

“*Entonces le damos un número de teléfono, esa persona va a poder comunicarse para decir ya empecé con el tratamiento, cómo se va sintiendo, qué síntomas de los esperados ocurre de los que ya conversamos y qué otros síntomas diferentes, que es mientras está usando el medicamento en la casa*”. (L. Córdoba)

“*Acompañamiento que mayormente hacemos dentro de la red de socorro en todo el país, tiene sus aristas particulares en un lugar donde las señales son muy complejas y que la gente vive en lugares muy alejados también, porque hay mucha comunidad rural, entonces eso también, ¿no? Hay mucha gente que vive en parajes o pueblitos*”. (A. Río Negro)

“*Pero sí, es una tarea que consume mucha dedicación y compromiso porque cualquier cosa que una diga en esa situación a veces angustiante o preocupante que está pasando la otra persona, por ahí puede aumentar esa angustia o esa preocupación*”. (A. Río Negro)

En este momento es clave la compañía y contención que las socorristas brindan desde el teléfono y la posibilidad que tienen de intervenir ante la posibilidad de alguna complicación. Todo el proceso que incluye la llamada, el taller y el momento del aborto sin duda constituye un “acompañamiento”. Pero en el momento del proceso de aborto en sí mismo, la presencia de alguien al teléfono según necesidad contribuye fuertemente a la seguridad del aborto con medicamentos en cuanto a la posibilidad de complicaciones y también al éxito en cuanto a la finalización del proceso con la expulsión completa.

En definitiva, este “acompañamiento” que implica el buen uso de la medicación, menos temores y estigmas, más confianza y seguridad contribuye a estandarizar el proceso, lo cual redunda en el bienestar de cada mujer que aborta en estas circunstancias y hace cada vez más seguro el procedimiento.

La estrategia de proponer a las mujeres que hagan una visita médica para anticoncepción y un control médico posaborto es vista por las organizaciones como una apuesta política para interactuar con el sistema de salud.

“*No, jamás dejamos de hablar, no es que se deriva, no, no se consigue un turno, se trabaja articuladamente*”. (M. Catamarca)

“*Por último, hacemos un seguimiento pos: cómo se sintió, si tuvo que recurrir a la guardia, si es así, cómo fue tratada, si fue a una guardia privada, si fue al sistema público, cómo la reciben*”. (N. Misiones)

La visita médica posaborto queda librada a la voluntad de cada mujer, pero siempre se cierra el acompañamiento con una llamada socorrista para completar los datos de finalización del proceso. Todo el proceso y los datos sociodemográficos anonimizados de las personas quedan plasmados en la sistematización^16^ que llevan adelante las socorristas, que son de carácter público y dan cuenta de las tareas llevadas a cabo y sus características.

#### Percepciones, sentimientos y satisfacción con la tarea

Se indagaron los sentimientos de las socorristas en dos momentos: en el primer contacto y al terminar el proceso. Para orientar la respuesta, se mencionaban algunas sensaciones que las entrevistadas podían seleccionar, ampliar, describir y profundizar. Las sensaciones que se listaban como opciones eran: *ansiedad, orgullo, cansancio, satisfacción, pena, empatía, incertidumbre y otras.* Las socorristas entrevistadas mencionaron la mayoría de las sensaciones listadas para describir los sentires en ese primer contacto. Ese momento fundante, en que las personas deciden interrumpir su embarazo y acuden al teléfono para establecer contacto con alguna integrante de la grupa, es constitutivo del acto de acompañamiento, y en los sentires mencionados se juega esa constitución. Las sensaciones más mencionadas fueron *empatía, ansiedad e incertidumbre*. La empatía surge como esa sensación de consustanciarse emocionalmente con el momento por el que pasa la persona que decide abortar, con la situación de vulnerabilidad que atraviesa y también con el valor y la fuerza que la moviliza a tomar la decisión:

“*En general es empatía el sentimiento. Pienso en la necesidad que las mueve y eso, es como que ellas están, la mayoría están solas, la mayoría no tienen recursos, o las adolescentes, están como todo ante la inminencia y el gran problema que les va a cambiar su vida, que las va a arruinar. Como decir bueno, la empatía ante esa situación de horror que la mayoría expresa, eso es como en general el sentimiento*”. (M. Corrientes)

En las respuestas que seleccionaron *empatía* también aparece *ansiedad e incertidumbre como* sensaciones asociadas al momento en que se establece el primer contacto y comienzo del vínculo con la persona que solicita el socorro. La *ansiedad* se explicita como un sentir que moviliza inseguridades, imaginarios, trayectorias y características de personalidad de las socorristas que se manifiestan en ese momento inicial. Junto con la *incertidumbre* dan cuenta de la intensidad y el compromiso que hace al dispositivo y a la configuración de red.

“*Primero que todo, ansiedad. Siempre me da mucha ansiedad poder hacer todo. Como que no esperen, que no, bueno, que no pasen tiempo, que no se sientan solas, que no sientan que no estoy respondiendo*”. (N. Ciudad Autónoma de Buenos Aires)

“*Cuando recién empezaba, hace algunos años, sí me aparecía más ansiedad porque desconocía el procedimiento, porque también como muchos, como la mayoría de la sociedad, tenía también las mismas ideas o imaginario en relación con el aborto, entonces pensar que una mujer podía poner en riesgo su vida por un aborto, también era una idea previa que yo traía y que entonces me tomó tiempo sacarme eso para que la ansiedad fuese menor*”. (M. Río Negro)

La *incertidumbre* en la expresión de las entrevistadas refleja también lo que sienten ante la diversidad de contextos y situaciones que relatan las personas que solicitan el acompañamiento. Algunas entrevistadas asocian momentos de mayor incertidumbre cuando el acompañamiento lo solicitan adolescentes.

*Satisfacción* y *orgullo* fueron sensaciones mencionadas en muchas de las entrevistas, y revisten un sentido de pertenencia, de afirmación política, de práctica feminista. El orgullo invade el momento del primer contacto, porque las socorristas sienten y piensan que, una vez más, el acontecimiento de ese llamado, de ese contacto afirma la posibilidad genuina de decidir un proyecto de vida sin mandatos, sin violencias. Es resistencia, es la posibilidad de quebrar el orden patriarcal, y la satisfacción emerge en ese sentir y en esa comprensión que también comporta aprendizajes compartidos:

“*Una sensación recurrente es de satisfacción. Es como una situación, es como una sensación de satisfacción de haber podido, cada aborto que se acompaña es como la satisfacción de haber podido participar de un acompañamiento donde no solamente una persona decidió sobre su vida, sobre su proyecto de vida y su futuro también, ¿no?*”. (M. Entre Ríos)

A las sensaciones de *satisfacción y orgullo* se suman, de forma espontánea, *alivio, tranquilidad, alegría, entusiasmo y agradecimiento*, que pueden encuadrarse en la opción “otras”. Todas las mencionadas fueron expresadas por las socorristas, independientemente de las listadas en la pregunta estructurada, para describir lo que activa ese primer contacto:

“*Prevalece esto del entusiasmo, de acompañar a una más, que va a comprender que está en su legítimo derecho de decidir sobre cuántos hijos criar, cuántos parir o no*”. (M. San Luis)

“*Alegría un poco de poder ayudar, pero también de la red que se genera. Siempre las mujeres que se comunican son porque entablaron contacto con alguien más, es una amiga que les pasó el número, es una prima, es la mamá, es la hija, digamos como eso también. Y cada llamado es porque el número se difundió de alguna forma, entonces está llegando y eso es bueno*”. (S. Chubut)

En el final del proceso, todas las percepciones y sentimientos de las socorristas son sentimientos positivos. La satisfacción, alegría, alivio, felicidad, orgullo devienen, según sus respuestas, de cuestiones relacionadas con la propia existencia de este activismo feminista tan especifico, el activismo de llevar a la realidad una práctica virtuosa. Algunas de las siguientes frases plasman lo dicho:

“*Me parece que cada aborto que nosotras garantizamos, un aborto cuidado, un aborto seguro digamos, es como que es una víctima menos de la morbimortalidad materna*. (Y. San Juan)

“*Somos todas más libres a partir de que una decide también, porque esa decisión se replica después*”. (C. Río Negro)

“*Para mí es súper importante saber que estamos celebrando algo, que no estamos sufriendo algo*”. (M. Neuquén)

“*O sea, cómo producir un cambio en la vida del otro y que el otro te cambie la vida a vos también*”. (N. Ciudad Autónoma de Buenos Aires)

“*Me quedo muy tranquila al saber que hubo una persona que pudo decidir y que lo hizo de forma segura, sin arriesgarse a nada inseguro y lejos de todo maltrato*”. (M. San Luis)

“*Siento alivio, me siento de alguna manera empoderada también de haber tenido la posibilidad de ayudar a alguien*”. (M. Salta)

“*Nos da orgullo que hayan tomado esa decisión, que hayan roto tantísimas barreras, porque nos llaman mujeres de 40 años en situaciones totalmente violentas, escondiéndose del marido, yendo básicamente contra viento y marea y toman la decisión igual y lo hacen a escondidas y se aguantan los dolores para que nadie se dé cuenta. Son situaciones muy fuertes a veces las que llegan y te da orgullo que hayan tomado esa decisión y que es su vida*”. (P. Tierra del Fuego)

Hablan del agradecimiento que les transmiten las mujeres. Además, se rescatan las cosas aprendidas en esa interacción:

“*Ese aprender la escucha y la paciencia como dos herramientas que necesito para el socorro y darme cuenta de que esas dos herramientas son para la vida, para todo. Que siempre es con otras*”. (A. Neuquén)

“*Acompañamiento en situaciones de aborto te sirve para el crecimiento personal y poder vos ver tus capacidades de vincularte con las otras personas porque es un vínculo muy íntimo*”. (N. Formosa)

“*La sociedad nos deja muy solas y que falta muchísimo para aprender a acompañar en la dimensión que nos merecemos, y que también nos falta trabajar, dejar acompañar y eso entre todas. ¿No? Entonces, creo que hay una apuesta que es infinita hacia la ternura, la ternura nos hace muchísimo más fuertes, muchísimo más poderosa y desde ese lugar siempre me interesa encarar, trabajar y vislumbrar*”. (E. Buenos Aires)

#### Relación con el sistema de salud (público y privado) y sus proveedores

Hay dos situaciones del vínculo con los sistemas de salud formal que han sido descriptas en las entrevistas y que, como ya dijimos, son una apuesta política del socorrismo para generar una articulación con el sistema de salud. Una de ellas es el cuarto paso del proceso, el posaborto, en el que se anima y promueve que las mujeres que abortaron concurran al sistema de salud para recibir cuidados médicos, si fueran necesarios, y anticoncepción posaborto.

La otra apuesta de articulación se relaciona con ofrecer, durante el primer contacto en la llamada, la opción de concurrir al sistema de salud para obtener un aborto, en el contexto de la Interrupción Legal del Embarazo (ILE). Al momento de realizar el estudio, la ILE era el único aborto permitido en Argentina, y podía llevarse a cabo, de acuerdo con las excepciones del Código Penal, en los casos en que hubiera peligro para la vida o la salud de la gestante o en un embarazo por violación. Se ofrecía, sobre todo, a aquellas mujeres que, cuando llamaban, decían que tenían una causal de aborto, como una violación o una condición de salud física severa, y en el imaginario médico hegemónico deberían ser imposible de rechazar.

La característica más sobresaliente de ese vínculo descripto por las entrevistadas refiere a la palabra “depende”, y remite a la diversidad de ámbitos de salud, de provincias y de efectores del equipo de salud y del devenir del contexto histórico. Lo que prima es la diversidad en las relaciones y la falta de arraigo en el territorio de los equipos de salud que garantizan la práctica.

Los hitos históricos, como el fallo F.A.L. y los sucesos de la “Marea Verde” y la aprobación en diputados de la Ley de Acceso a la Interrupción Voluntaria del Embarazo en 2018, se sumaron a las políticas públicas que esto generó, como la aprobación por parte de la Administración Nacional de Medicamentos, Alimentos y Tecnología Médica (ANMAT) de la comercialización del misoprostol de 200 mg y el protocolo del Ministerio de Salud de la Nación. Estos hitos mejoraron en muchos casos el contexto y las relaciones y articulaciones hacia adentro de las instituciones de salud y con las socorristas. 

En los vínculos se visualizan acciones concretas, como orientar la demanda hacia servicios, no maltratar a mujeres que habían abortado, como muestran los siguientes relatos:

“*Lo que también sucede es en esta cuestión de que nosotras sabemos a quién derivar. Entonces, si yo me baso en eso, te puedo decir superfluido y amigable, pero hay un trabajo previo de saber, con quién*”. (E. Buenos Aires)

“*Y que sí más intentamos como ajustar ahí el filtro para que las mujeres no lleguen a los lugares donde son más expulsivos o maltratadores, sino que intentamos tener recurseros precisos, con información clara de con quién, haber acordado antes con el área médica*”. (E. Buenos Aires)

“*Creo que sí es una decisión política cuidar ese vínculo desde las socorristas yo lo siento al vínculo que tenemos complejo, sinuoso, es difícil, pero lo estamos haciendo. Lo intentamos*”. (D. La Rioja)

La apuesta de creación y sostén del vínculo se deja ver también en la articulación no solo con equipos en el territorio, sino con referentes provinciales y funcionarios de los sistemas de salud, como expresan algunas entrevistadas:

“*Directamente hablamos con la jefa del departamento, pero la realidad que vive el ministerio es muy distinta a lo que se vive cotidianamente en el hospital y los centros de salud*”. (B. Río Negro)

Exigir y acompañar el reclamo de que la interrupción legal del embarazo (ILE) se resuelva en el sistema de salud tiene que ver con tratar de encauzarla a través de los equipos existentes en las instituciones de salud formal, o de pensar en el socorrismo como una salida elegible entre las dos posibles: el sistema de salud o las socorristas.

“*Cuando atendemos la línea siempre la primera cosa que se les informa a las mujeres es si ella sabe que existe la interrupción legal del embarazo, que ella tiene derecho a ir a solicitarla, si vive acá*”. (A. Río Negro)

Es importante considerar aquí las particularidades de nuestro país y su sistema de salud fragmentado, además de su extensión y su diversidad cultural. Hay enormes diferencias entre los sistemas de salud provinciales, las realidades, e incluso los marcos normativos y las culturas institucionales. 

No se puede cuantificar las respuestas en los términos que fueron planteados en la guía de entrevistas -por ejemplo, si el vínculo es operativo, fluido, amigable, sinuoso, expulsivo, etc.- porque la existencia o no de esta relación y su calidad depende de muchos factores, no solo de la zona del país, aunque esta influye a favor o en contra, como muy bien plantea la siguiente entrevistada:

“*No hay una respuesta única porque depende la zona. Por ejemplo, acá en esta zona donde estoy yo es expulsivo. El sistema nos expulsa, no acepta ningún tipo de articulación con socorristas y trata de que las socorridas que llegan por 0800 sin pasar por socorrismo*”. (D. La Rioja)

Una característica importante y que muestra las dificultades en la rectoría para el sostén de la interrupción legal del embarazo, es la imprevisibilidad y la precariedad en el sistema de salud de muchas provincias, con respecto a la provisión del servicio. El comportamiento de los equipos es errático y depende no solo de la zona del país, sino también del compromiso personal e ideológico y de la capacitación de los equipos.

La existencia en un determinado territorio de equipos que proveen interrupción legal del embarazo, o su sostenibilidad en el tiempo, depende de cuestiones que tienen que ver, por ejemplo, con la gestión del recurso humano médico, en algunos casos suelen quedar equipos desprovistos de personal idóneo y comprometido por renuncias o traslados, y deja de proveerse el servicio por tiempo indeterminado. Todo esto configura un panorama de inequidad en el acceso, que el socorrismo contribuye a remediar en muchas regiones, como plantean las entrevistadas:

“*Entonces es como una cosa media rara que el equipo funciona dependiendo a quién te toca*”. (N. Misiones)

“*Sí, centrado en las personas, claro, que cada uno decide si lo hace o no lo hace, y lo hace cuando está de humor y si no está de humor, se vuelve objetor de conciencia de un día para otro. Eso pasa todo el tiempo*”. (M. Corrientes)

“*Dejó de trabajar allí y no quedó nadie como para coordinar, entonces como que estamos ahí buscando a gente no tan amigable, medianamente amigable, para poder contactar para algunas situaciones como, por ejemplo, las guardias, que no se las violente a las chicas, pero no hay más que eso*”. (D. La Rioja)

“*Había una profesional que era la que lo hacía más operativo y amigable, pero se fue del sistema público, y aún por lo menos no hemos logrado otro vínculo*”. (M. Catamarca)

Las socorristas suelen ser críticas de los saberes médicos sobre el aborto, de la información que ofrecen y de los procedimientos utilizados en los servicios de salud para realizar interrupciones legales del embarazo. Argumentan que, en muchas ocasiones, los equipos de salud institucionales no conocen las dosis de misoprostol y que los procedimientos suelen fallar y terminar en abortos quirúrgicos:

“*Pero están haciendo las ILE o sea, están haciendo cualquier método pero los hacen. Empiezan con ‘miso’ y terminan en legrados y así está funcionando el equipo ILE*”. (M. Chaco)

En muchos casos, las socorristas resultan en docentes de los médicos en cuestiones técnicas referidas, sobre todo, al aborto con medicamentos:

“*Esta doctora, que le costó un montón decirnos que habían aprendido con nosotras a hacer abortos, que aprendieron un montón y que se animaron también después de vernos*”. (B. Río Negro)

Otro de los problemas que ven es que los equipos que garantizan la interrupción legal del embarazo solo indican tratamientos, pero no tienen empatía con la situación, como se plantea a continuación:

“‘*Vení, te doy las pastillas’, ahí no hay explicación, no hay una empatía, no hay una explicación del proceso, si va a doler, si no va a doler, cuestiones por ahí emocionales que transcurren en ese momento no hay nada*”. (M.E. Buenos Aires)

“*Después no sé, es como que a veces hay un afán por hacer sentir culpable a la persona, no sé. Dicen cosas innecesarias*”. (P. Tierra del Fuego)

El vínculo con el sistema privado y de obras sociales tiene diferencias con el descripto para el sistema público. En algunas jurisdicciones, muy pocas, las socorristas tienen relación con los dos subsistemas, pero siempre describen que la relación con el subsistema privado y de obras sociales es difícil o escasa:

“*Puedo hablar de una sola profesional que está en el sistema privado y eso contestaría la pregunta*”. (N. Misiones)

En esta relación se dejan ver algunas particularidades que no estaban en las descripciones del sistema público, por ejemplo, que es muy común que, desde estos consultorios privados, se les dé el número de las socorristas o se las envíe al hospital público a mujeres que quieren abortar desentendiéndose del proceso:

“*Cuando las mujeres van y les dicen que creen que están embarazadas y quieren abortar, automáticamente les pasan nuestro número, te sacan el tema de encima y no se garantizan ILE en el sistema privado*”. (M.E. Entre Ríos)

“*Nosotros no podemos. Andá al hospital*”. (E. Buenos Aires)

“*No les dan la información a las personas, pero les dan nuestro número*”. (E. Buenos Aires)

Reiteraron en varias ocasiones el mal manejo de la medicación que observan cuando se realiza una interrupción legal del embarazo en el sistema privado, dando cuenta del desconocimiento de los protocolos nacionales y de la OMS:

“*Y bueno este médico hacía un procedimiento pésimo, nosotras llevábamos a las mujeres a que se hagan esos procedimientos, mal, mal. Aplicaba dos vaginal, dos oxaprost vaginal, dos sublingual y le pedía que se pongan un tampón*”. (D. La Rioja)

“*Les dice, por ejemplo, que usen solamente cuatro misoprostol o seis, entonces por ahí les provoca un sangrado, pero es un procedimiento incompleto y él se asegura que esa persona vuelva a la clínica y le haga un legrado, así lo cobra*”. (M. San Luis)

A les mediques del sistema privado más colaborativos se los describe sobre todo proporcionando ecografías y controles posaborto:

“*Es un centro de diagnóstico por imágenes que lo que hacen es hacer ecografías sin orden del médico, entonces eso nos facilita muchísimo*”. (G. Buenos Aires)

“*Y van al posaborto, a hacerse el control posaborto y le dicen directamente en la cara que abortaron y cómo abortaron*”. (N. Misiones)

#### La pandemia

A partir de la situación de aislamiento por la pandemia de covid-19 surgió la necesidad de incluir en la entrevista una pregunta sobre cómo habían cambiado los procesos del socorrismo con la pandemia, en la que las mujeres están enfrentando dificultades únicas y diferentes.

En el contexto general de la vida de las mujeres, tanto socorristas como socorridas, se pueden ver: imposibilidad para trasladarse, para reunirse y para concurrir a servicios asistenciales y a esto se agregan las historias personales, como los cambios en las tareas del cuidado, las convivencias y las dificultades económicas, la necesidad de contar con conexión a Internet o con datos en el teléfono con lo que las dificultades fueron muchas y diversas.

Lo primero que relatan en la mayoría de las jurisdicciones fue cómo tuvieron que cambiar algunas lógicas y articular con el sistema de salud. La articulación con el sistema de salud se plasmó a través de la línea telefónica nacional gratuita y confidencial (el 0800-Salud Sexual) para brindar información sobre salud sexual y reproductiva de la Dirección Nacional de Salud Sexual y Reproductiva (DNSSR).

El llamado a esta línea lo realizaba una grupa socorrista o una mujer que, luego de haber llamado a las socorristas, estas le solicitaban que llame a la línea. Este llamado disparaba una “secuencia” desde la DNSSR, es decir, un mecanismo de comunicación con la referente provincial del programa, para proveer la medicación y un equipo del sistema de salud que se hiciera cargo del caso y estuviera en contacto con las socorristas. Desde SenRed se le daba igualmente el taller con la información y se le ofrecía acompañamiento durante el proceso. Las socorristas lo vieron como una articulación virtuosa según las opiniones vertidas en las entrevistas:

“*Nos sorprendió y nos sirvió mucho, una fue la estrategia de llamar… de derivar los llamados al 0800 de salud sexual y bueno, la verdad que eso acá nos viene funcionando rebien*”. (C. Río Negro)

“*Tratar de que todos esos abortos, en este contexto de pandemia, se resuelvan por salud pública*”. (M. Salta)

“*Bueno, te vamos a acompañar en todo este proceso, dure lo que dure, vamos a hacer que el sistema de salud responda. ¿Estás dispuesta a eso?*”. (E. Entre Ríos)

Los primeros momentos del aislamiento social preventivo y obligatorio se relatan como un momento de intensa incertidumbre para enfrentar la tarea en un contexto de aislamiento para ambas protagonistas, socorristas y socorridas:

“*En ese momento fue puro pánico y ahí sí se aceitó esta cuestión de empezar a utilizar el 0800 con más frecuencia para aceitar ahí los canales de diálogo*”. (M. Salta)

Hubo un consenso nacional y se decidió que las llamadas se gestionarán desde la red para que resuelvan a través de los efectores de salud pública, con interacciones y articulaciones ya existentes entre las socorristas y el sistema de salud o desde un llamado a la línea 0800 de salud sexual y reproductiva.

“*Y coordinamos con los efectores de salud de acá de…, son tres ahora, antes era uno solo, para que las mujeres accedan a ellos y les den pastillas. Y después nosotras hacemos todo el seguimiento y el acompañamiento*”. (K. Buenos Aires)

El nuevo funcionamiento se informaba en la primera llamada, los talleres siguieron haciéndose en forma virtual por teléfono, WhatsApp u otra herramienta de videollamada según la región y las necesidades:

“*Ya no podemos hacer los talleres grupales, que para nosotras eso es algo que es un cambio sustancial que al principio lo sentimos mucho, porque hay mucha cosa en ese encuentro grupal, pero a medida que fue pasando el tiempo le encontramos otra vuelta a la cuestión de estas nuevas formas de encontrarnos con las personas que vamos a acompañar*”. (M. Río Negro)

Esta nueva forma, si se la piensa como única alternativa viable en tiempos de pandemia, presenta múltiples dificultades como se relata a continuación:

“*Los talleres, al principio, intentamos hacerlos grupales por la virtualidad a quienes tenían buena conexión ¿no? Que podían conectarse, bueno aparecieron otras preguntas, ¿tenés wifi en tu casa o usas datos?*” (N. Formosa)

En el contexto de pandemia, relatan que hubo que pensar en situaciones, relacionadas con la vivienda y la convivencia, en las comunicaciones y en las relaciones de las mujeres con su entorno y con el sistema de salud para poder pensar en el momento y lugar específico del aborto con medicamentos:

“*El hacinamiento, el hacinamiento es tremendo y por ahí tenemos personas que están embarazadas y nadie en la familia sabe y en ningún momento están solos o solas, entonces hay que esperar… es más, hemos dado talleres a las 3 de la mañana, a las 5 de la mañana, a las 12 de la noche, a las 2 de la tarde*”. (M. Salta)

“*Tuvimos muchos acompañamientos a la noche de mujeres que lo tenían que hacer como más a escondidas para que no se enterara la familia*”. (C. Catamarca)

“*Sí las acompañamos a pensar estrategias, a pensarlas con todo lo que implica ocultar a la familia, entonces están las amigas, ayudamos a recuperar esos vínculos que a veces las mujeres creemos que estamos solas, y en la desesperación: ‘No. No hay nadie, no hay nadie’. Entonces eso, acompañamos a recuperar esas lealtades que sabemos que las tenemos y sabemos que nos van a acompañar*”. (M. San Luis)

“*Ahora es como que la escucha atenta se ha vuelto casi vital digamos, también para hablar de los miedos que existen, por ejemplo, al ir al hospital, en medio de esta incertidumbre que genera la pandemia*”. (L. Córdoba)

“*Y además se han intensificado los acompañamientos. Son muchas horas, porque el aislamiento, que potenció todas estas sensaciones y emociones de angustia, incertidumbre, las que sufren violencia de género, las personas que se han quedado sin trabajo, entonces se han intensificado, entonces duran mucho más, muchas horas, mucho más tiempo de charla*”. (M.P. Buenos Aires)

#### El socorrismo y la Ley de IVE/ILE

A pesar de que todas las entrevistas fueron realizadas antes de diciembre de 2020, cuando se votó la Ley de Interrupción Voluntaria y Legal del Embarazo, en la guía de entrevistas ya se había previsto preguntar a las lideres de La Revuelta qué roles pensaban que deberían asumir como socorristas ante la legalización del aborto.

Primariamente, el dispositivo socorrista fue encarado como una actividad que estaría vigente hasta que el sistema de salud asuma por ley su deber de realizar las interrupciones legales del embarazo.

“*En el año 2010 que armamos el espacio de Socorro Rosa, siempre el interés político fue porque desde siempre pensamos que las socorristas íbamos a dejar de existir cuando estuviera la ley, éramos como el plan B*”. (R. Neuquén)

Sin embargo, dado el tiempo transcurrido, el trabajo realizado, sistematizado y publicado, más la experiencia acumulada, los aprendizajes, las relaciones, las articulaciones y los múltiples crecimientos y entrecruzamientos con el devenir histórico, estas ideas primarias fueron cambiando y desde hace algunos años, la apuesta es otra:

“Acompañar a las mujeres y a otras personas que necesitan abortar desde nuestro activismo como revueltas socorristas implica poner a disposición una política de cuidados alentada por la ética feminista en la que los cuidados son siempre colectivos”. (R. Neuquén)

“Los sostenes emocionales para no desalentar la decisión tomada, para que esa decisión se viva como parte de la justicia, la dignidad y la vida elegida, sin miedos, ni vergüenzas, ni riesgos para la salud presente ni futura”. (R. Neuquén)

“La creación de caminos sin laberintos burocráticos, sin violencias, ni estigmas, ni culpabilizaciones, sin ceños fruncidos ni cejas levantadas”. (R. Neuquén)

“Nosotras aspiramos a que cuando esté la ley se pueda incluso reglamentar las posibilidades de acompañamiento por fuera del sistema de salud. Y que entonces sea la persona que va a abortar quien, sabiendo todas las posibilidades que tiene, elija. Y decida”. (M. Neuquén)

“Miremos más los límites que tiene el sistema de salud, pero también las posibilidades de que ofrece el socorrismo, comparemos y dejemos a las mujeres elegir”. (R. Neuquén)

## DISCUSIÓN

Este estudio se programó y desarrolló antes de la existencia de la Ley 27610 de Interrupción Voluntaria y Legal del Embarazo, entre los años 2019 y 2021. En ese periodo ya había un camino recorrido en Argentina con relación al aborto: el fallo F.A.L. de la Corte Suprema de Justicia de la Nación sentó las bases para implementar el aborto permitido por las excepciones del código penal en el sistema de salud, y abrió caminos de autogestión y procesos comunitarios.

También la presentación del proyecto de Ley de Interrupción Voluntaria del Embarazo en 2018, que fuera ampliamente discutido y aprobado en la Cámara de Diputados de la Nación, y rechazado en el Senado de la Nación, amplió la conversación social y permitió mejorar las políticas públicas existentes y disminuyó el estigma y la clandestinidad.

El accionar socorrista nace en la provincia de Neuquén en el 2012 creado por la colectiva feminista La Revuelta y en 2014 se crea la red nacional Socorristas en Red (SenRed). Toma entonces mucha relevancia el trabajo de las organizaciones de la comunidad, acompañando procesos de aborto en un contexto de restricción legal, donde existía el temor a la penalización y estigma social sobre la práctica y escasa resolución de abortos por parte del sistema de salud formal/institucional.

Los hallazgos de este estudio dan cuenta de las motivaciones e historia de la colectiva feminista La Revuelta de la provincia de Neuquén, en Argentina, teniendo en cuenta los hitos y momentos claves de su devenir histórico: cómo se llega a la creación e implementación del dispositivo “Socorro Rosa” y cómo las reflexiones sobre la práctica van modelando un dispositivo único y singular llamado “acompañamiento”.

También, como se crea y desarrolla la red nacional Socorristas en Red, quiénes son sus integrantes, cuáles son sus motivaciones para llevar adelante la tarea, las emociones y sentimientos que esta les provoca, sus relaciones con otras organizaciones comunitarias, políticas e institucionales, cómo se gestaron las relaciones con el sistema de salud y cómo se desarrollan actividades de sistematización de datos y de producción y difusión de conocimientos.

Los hallazgos en relación con la red nacional Socorristas en Red aportan una descripción y caracterización en profundidad de una experiencia comunitaria y colectiva del activismo feminista argentino, de las personas que la componen, de sus motivaciones, de sus singulares y creativos métodos y de sus relaciones con la comunidad y con el sistema de salud. En el relato de la historia, se destaca la definición de la agrupación feminista La Revuelta, situándola dentro de un feminismo de la calle y de la acción. Se definen interesadas en la política en general, pero no solo para “mirarla y opinarla”, sino para intervenir.

Entre los hitos históricos, resaltamos aquellos que mueven hacia adelante y consolidan las prácticas feministas en las que nace el socorrismo, por ejemplo, la incorporación de la agrupación La revuelta a la Campaña Nacional por el Aborto Legal, Seguro y Gratuito. En el inicio y difusión del socorrismo sobresalen las creativas estrategias para resolver problemas prácticos para poder proveer la medicación y para consolidar las relaciones con el sistema de salud y el hallazgo novedoso de un sistema de salud local (como el de Neuquén) tanto público como privado, con apertura y disposición para articular. Asimismo, resulta destacable y virtuosa la estrategia de registrar los datos, que permite tomar decisiones operativas según los resultados obtenidos, y las prácticas de docencia e investigación articuladas con universidades y sistemas de salud de la zona de origen.

En cuanto a la red de socorristas se plasman sus sentimientos y vivencias personales, así como las motivaciones para unirse, participar y gestionar la tarea, también sus sensaciones y sentimientos a lo largo y al final del proceso que nos permiten entender la potencia y belleza de su trabajo y la sustentabilidad en el tiempo del dispositivo. Las motivaciones para sumarse y los sentimientos y sensaciones son de raíz feminista y están atravesadas por experiencias personales de aborto o militancias políticas y un fuerte deseo de “hacer” para transformar una realidad que las incomoda. Su accionar contempla siempre emociones, las emociones que dan cuenta de la intensidad y el compromiso que hace al dispositivo y a la configuración de red. Una red de acompañamiento que emerge desde los saberes y conocimientos conquistados en la experiencia y que, como construcción colectiva, no está exenta de marchas y contramarchas, preguntas, miedos y batallas que se condensan en esas sensaciones. 

El otro tipo de indagaciones a las socorristas fueron más puntuales y descriptivas sobre el proceso de acompañamiento y sus relaciones con el sistema de salud público y privado de la región en la que actuaban. El dispositivo va mucho más allá de la oferta de información, redefine los cuidados para las mujeres que abortan, lo llaman “acompañamiento” y lo definen con sus propias palabras como: poner a disposición una política de cuidados alentada por la ética feminista en la que los cuidados son siempre colectivos y la consideración de que quien aborta es la protagonista y dueña de todo el proceso. Esto se basa en la disponibilidad para dar tiempo propio, para estar con el teléfono encendido para quien quiera abortar a la hora en la que decide abortar, para repasar indicaciones, para calmar dolores, para agregar consejos, sugerencias, para sugerir profesionales amigables donde acudir. Poniendo hincapié en que lo que le pasa a esa mujer les importa, las conmueve y las mueve a colaborar para encontrar un camino de resolución que sortee los obstáculos y las angustias. Armar un sostén emocional para no desalentar la decisión tomada, para que ésta se viva como parte de la justicia y la dignidad de la vida elegida, sin miedos ni vergüenzas ni riesgos para la salud, y armar caminos sin laberintos burocráticos, sin violencias y sin estigmas.

Los hallazgos de este estudio confirman y amplían la bibliografía existente y la conversación actual sobre la autogestión del aborto y la intervención de las agrupaciones feministas en el acompañamiento de estos procesos. Existe un creciente cuerpo de conocimientos que abona a la redefinición de los cuidados en el aborto, esto implica una evolución y expansión de las prácticas y los enfoques en la provisión de servicios de aborto. En sus aspectos claves, además de los conceptos de salud integral, acceso universal y equitativo, despenalización y derechos humanos, atención centrada en las necesidades de las personas y basadas en evidencia, se encuentran particularmente los conceptos de integración con otros servicios de salud y desmedicalizacion, que enmarcan puntualmente el trabajo feminista y colectivo de las socorristas que caracterizamos en este estudio^17^. 

Desde el advenimiento y la generalización del uso de medicamentos para abortar, las mujeres pueden manejar sus propios abortos con pequeña o ninguna supervisión o ayuda y con adecuada información^18^. También sabemos que, en forma masiva o abrumadoramente superior, eligen tener sus abortos en su casa, logrando control y privacidad en todo el proceso ^19^^,^^20^^,^^21^^,^^22^^,^^23^. 

Las organizaciones de la comunidad, en general organizaciones feministas, que a través de líneas telefónicas específicas o plataformas en la web u otros recursos ayudan a las mujeres a abortar, han emergido alrededor del mundo como fuentes de información, con una propuesta diferente a la atención médica, ofreciendo un dispositivo de soporte confiable, desinteresado y generoso durante el proceso de aborto^13^.

La bibliografía señala que estas organizaciones comparten en general algunas características: la desmedicalizacion, utilizando el conocimiento y la tecnología médica desde un contexto comunitario, haciéndolo accesible, el cambio en las relaciones de poder entre las poblaciones sin acceso y el sistema de salud formal institucional, lo cual incrementa la autonomía y autodeterminación, además de su disposición a trabajar en los límites de la ley cuando es necesario^24^. 

Estas experiencias y modelos de facilitación de la autogestión del aborto en el mundo, son un tema recientemente abordado por la literatura médica^23^^,^^25^ y, en este momento, forma parte del diálogo social sobre el acceso al aborto, que incluye preguntas sobre ¿quiénes componen estas organizaciones? ¿cuáles son sus métodos y cuál es su impacto socio sanitario?^13^^,^^26^^,^^27^; ¿cómo son y cuán seguras y efectivas son estas intervenciones y dispositivos de atención?^28^; ¿cómo y por qué las mujeres prefieren tener sus abortos fuera del sistema formal de salud, aun en los contextos donde es legal?^29^; ¿cuáles son las percepciones y experiencias de las mujeres y cuan aceptables y satisfactorias resultan estas experiencias?^30^.

Respecto al vínculo con el sistema de salud, la bibliografía abona a algunas de las descripciones de las socorristas, reportando acceso dificultoso, estigmatización hacia la práctica del aborto, baja calidad de atención y discriminación por parte de los sistemas formales de salud hacia las mujeres que abortan y hacia las organizaciones de la comunidad que colaboran con la autogestión del aborto^12^^,^^19^^,^^20^^,^^22^^,^^23^^,^^31^^,^^32^. 

El camino recorrido en este estudio nos lleva a pensar que, en términos simbólicos, la definición del modelo comunitario de acompañamiento de abortos reserva esta palabra para el modelo del socorrismo, el acompañamiento no es lo mismo que la atención médica que se desarrolla en el sistema institucional de salud, aunque esta sea integral, adecuada, centrada en las personas y ajustada a los derechos de las mujeres. Es un modelo alternativo y desmedicalizado de atención, que tiene otras características. Es factible, seguro y efectivo y está validado por la evidencia^33^^,^^34^^,^^35^.

Actualmente estos cuidados comunitarios han sido incluidos en la guía de OMS “Directrices sobre la atención para el aborto”^17^, publicada en 2022. Dentro de los enfoques de prestación de servicios de aborto, la guía incluye el modelo de acompañamiento comunitario y de autogestión y también, al hacer referencia a la llamada estrategia de ampliación de roles o tareas compartidas para los cuidados del aborto, incluye a las organizaciones de la comunidad como miembros activos del equipo de salud^17^.

En este sentido la bibliografía plantea que actualmente, para algunos cuidados en salud, existe, por un lado, un sistema médico o un sistema de salud formal en el que los cuidados ocurren bajo un control institucional y, por otro, un sistema de salud autónomo con formas de activar ancladas en la justicia social y que trabaja en contextos comunitarios como sería el caso de las Socorristas en Red (SenRed) de Argentina^24^.

En este estudio, la descripción del proceso da cuenta de prácticas feministas colectivas, que tienen el componente de escuchar, desestigmatizar y acompañar decisiones de otras. A su vez, son prácticas que están previamente acordadas en la red, se ofrecen por escrito, están sistematizadas, basadas en evidencia y son sencillas y adaptadas a la realidad local, centradas en la persona, accesibles fácilmente, factibles, efectivas y seguras. 

En cuanto al vínculo con el sistema de salud formal o institucional, que caracteriza a las Socorristas en Red de Argentina descripto en nuestro estudio y mostrado también en otros estudios^36^^,^^37^, es proactivo desde las socorristas hacia el sistema de salud institucional, y comenzó centrado en la necesidad de recurrir si hay complicaciones o para situaciones pendientes en el posaborto y la anticoncepción. Ese vínculo deseado y estimulado no es fácil, y es una construcción que, desde Neuquén, las socorristas de La Revuelta llevaron adelante, lo promovieron en la red nacional y lo sostienen en la actualidad. Según las indagaciones de este estudio, existen vínculos y articulaciones virtuosas que se retroalimentan desde hace años y que alcanzan a equipos en el territorio y también a referentes provinciales y funcionarios de los sistemas de salud institucionales. 

Destacamos como particularmente virtuosa la transmisión al sistema de salud institucional de las décadas de experiencia en el uso del misoprostol y en el seguimiento que implica el proceso de acompañamiento de las socorristas. Ese proceso se realiza a través de instancias formales como seminarios y cátedras universitarias y capacitaciones a residentes que, sin dudas, favorecieron al sistema de salud y son bienvenidas y aceptadas socialmente. En esa relación, también, se genera un vínculo bilateral que beneficia el acceso de las mujeres^38^.

Sin embargo, cuando la red se extiende a todo el país los resultados de este vínculo son dispares, y en algún sentido tiene que ver con las características del sistema de salud argentino que está fragmentado en subsectores y a su vez es autónomo y diferente en cada provincia. Eso genera una gran diversidad de respuestas del sistema de salud formal al accionar socorrista y a sus intentos de articulación^36^^,^^37^.

Resulta muy importante de resaltar el aporte de datos sociodemográficos de las mujeres que abortan acompañadas por el dispositivo socorrista en argentina, sobre todo, por dos razones: la primera, porque en la situación de restricción legal los datos previos a la legalización son escasos y, la segunda, porque permite ver la magnitud del trabajo comunitario a lo largo y ancho de todo el pais. En un estudio comparativo de tres modelos existentes en Argentina, que proveían abortos antes de la Ley 27610, se visualiza claramente esta magnitud: en el año 2019, SenRed acompañó a abortar a 28.454 mujeres en todo el país y en el mismo periodo 8.868 abortos fueron realizados por proveedores de salud^36^^,^^39^.

La situación de aislamiento por la pandemia no solo afectó el estudio en lo operativo, sino que también afectó profundamente el accionar socorrista, la vida de las mujeres en general y mucho más la de aquellas que estuvieron en situación de aborto durante el aislamiento social obligatorio^37^^,^^40^^,^^41^. 

Las socorristas reconfiguraron el modelo de acompañamiento recurriendo a la telemedicina para los talleres y estrecharon las articulaciones con el sistema formal de salud a través de la línea de consultas 0800 de la Dirección Nacional de Salud Sexual y Reproductiva. Estos cambios se convirtieron en un sostén vital para las mujeres en situación de aborto, pero también permitieron mejorar y profundizar los lazos con el sistema de salud, haciendo visible la posibilidad de articulaciones virtuosas en pos de la mejora en el acceso al aborto.

Con la legalización y el escenario pandémico con las interacciones frecuentes y directas entre las referentes provinciales de salud sexual y reproductiva y las socorristas, se pone en valor la idea de que este trabajo comunitario que se viene haciendo no será reemplazado por el sistema de salud. 

## CONCLUSIONES

Las experiencias de acompañamiento a la autogestión del aborto con medicamentos por parte de organizaciones de la comunidad existen en Asia, África y Latinoamérica^34^^,^^35^ y han sido identificadas como una de las causas de la disminución de la morbimortalidad por aborto en contextos restrictivos y de la generación con su accionar de evidencia sobre su efectividad y seguridad.

Los hallazgos de este estudio ponen en valor una experiencia que, desde la Patagonia argentina, se convierte en una red comunitaria nacional. Esta red y su organización de origen, La Revuelta, tienen características que le son propias y su modelo de acompañamiento de abortos es singular. El dispositivo da cuenta de prácticas feministas colectivas, que tienen el componente de escuchar, desestigmatizar y acompañar decisiones de otras, a su vez, son prácticas que están previamente acordadas en la red, se ofrecen por escrito, están sistematizadas, basadas en evidencia y son sencillas y adaptadas a la realidad local, centradas en la persona, accesibles fácilmente, factibles, efectivas y seguras.

Particularmente, se detecta su crecimiento y su plasticidad metodológica para persistir en el tiempo, la dimensión numérica de su resolución de abortos, el aporte a la investigación y a la toma de decisiones de sus registros sociodemográficos, sus alianzas estratégicas con los sistemas de salud institucionales público y privado y con el sistema educativos y su producción en investigación y sus publicaciones. 

En definitiva, los resultados de este trabajo coinciden con la conversación y la producción bibliográfica internacional acerca de estas organizaciones y destaca sus fortalezas y la necesidad de incorporarlas a los cuidados de la salud del sistema institucional en nuestro país.
